# The Critical Role of Astragalus Polysaccharides for the Improvement of PPRAα-Mediated Lipotoxicity in Diabetic Cardiomyopathy

**DOI:** 10.1371/journal.pone.0045541

**Published:** 2012-10-01

**Authors:** Wei Chen, Yanping Xia, Xuelan Zhao, Hao Wang, Wenjie Chen, Maohua Yu, Yiming Li, Hongying Ye, Yu Zhang

**Affiliations:** 1 Department of Geriatrics, Hushan Hospital, Fudan University School of Medicine, Shanghai, China; 2 Department of Endocrinology, Huashan Hospital, Fudan University School of Medicine, Shanghai, China; 3 Core Center of Animal Facility, Fudan University School of Medicine, Shanghai, China; University of Western Ontario, Canada

## Abstract

**Background:**

Obesity-related diabetes mellitus leads to increased myocardial uptake and oxidation of fatty acids, resulting in a form of cardiac dysfunction referred to as lipotoxic cardiomyopathy. We have shown previously that Astragalus polysaccharides (APS) administration was sufficient to improve the systemic metabolic disorder and cardiac dysfunction in diabetic models.

**Methodology/Principal Findings:**

To investigate the precise role of APS therapy in the pathogenesis of myocardial lipotoxity in diabetes, db/db diabetic mice and myosin heavy chain (MHC)- peroxisome proliferator-activated receptor (PPAR) α mice were characterized and administrated with or without APS with C57 wide- type mice as normal control. APS treatment strikingly improved the myocyte triacylglyceride accumulation and cardiac dysfunction in both db/db mice and MHC-PPARα mice, with the normalization of energy metabolic derangements in both db/db diabetic hearts and MHC-PPARα hearts. Consistently, the activation of PPARα target genes involved in myocardial fatty acid uptake and oxidation in both db/db diabetic hearts and MHC-PPARα hearts was reciprocally repressed by APS administration, while PPARα-mediated suppression of genes involved in glucose utilization of both diabetic hearts and MHC-PPARα hearts was reversed by treatment with APS.

**Conclusions:**

We conclude that APS therapy could prevent the development of diabetic cardiomyopathy through a mechanism mainly dependent on the cardiac PPARα-mediated regulatory pathways.

## Introduction

Diabetes mellitus affects more than 180 million people around the world, and this number is anticipated to increase to 300 million by 2025, within which type 2 diabetes might account for 90–95% [1]. Cardiovascular disease is by now the leading cause of morbidity and mortality in diabetes. Although the underlying causes are multifactorial, the importance of cardiac dysfunction independent of coronary artery disease or hypertension, a condition termed as diabetic cardiomyopathy, has now been further emphasized [2], [3].

Growing evidence suggests that altered myocardial substrate and energy metabolism mainly contribute to the development of diabetic cardiomyopathy [4]. The heart uses a variety of substrates for energy production, including fatty acids (FAs) and glucose. The diabetic heart is characterized by reduced glucose metabolism and enhanced FA utilization [5]. Data from several diabetic animal models indicated that diabetic hearts had elevated rates of FA oxidation, ectopic fat deposition and subsequent lipid peroxidation by peroxisome proliferator-activated receptor (PPAR) α regulatory pathways, leading to lipotoxic cardiomyopathy, which finally resulted in ventricular dysfunction [6], [7]. Metabolic modulation to repress lipid oxidation and sustain glucose use in myocardium appeared to prevent cardiac dysfunction in models of severe type 2 diabetes [8], [9]. Similar results were observed in human patients with type 2 diabetes, suggesting that pharmacological interventions that reduce cardiac FA utilization may improve cardiac performance [10], [11].

Astragalus polysaccharide (APS), is one of the main active ingredients of astragalus membranaceus, a traditional herbal Chinese medicine. The polysaccharidesare a mixture of APS I and II. APS I is a kind of heterosaccharide which is composed of D-glucose, D-galactose, and L-arabinose in molar ratios of 1.75∶1.63∶1, with the average molecular weight of 36.3 kDa. APS II is a kind of dextran with high molecular weight, bonded mainly with a-(1→4)-D-glycosidic linkages [12]. Our previous study showed that APS administration strikingly improved the systemic metabolic disorder in STZ- induced diabetic mice, with the reversal of hyperglycemia and hyperlipidemia. Moreover, treatment with APS also ameliorated cardiac dysfunction and well-protected myocardial ultrastructure in diabetic hearts [13]–[15]. However, whether APS therapy has direct effects on diabetic cardiomyopathy, independent of its influence on metablic derangements systemically, is still unclear. To further explore the potential mechanism, we throw the hypothesis that APS may improve diabetic cardiomyopathy with the inhibition of myocardial lipotoxicity dependent on the cardiac PPARα regulatory pathway. Transgenic mice with cardiac- restricted over-expression of PPARα (myosin heavy chain [MHC]-PPARα mice) exhibit a cardiac metabolic phenotype which is strikingly similar to that of the diabetic heart with increased FFA utilization and decreased uptake and oxidation of glucose, presenting features of lipotoxic cardiomyopathy without hyperlipidemia or hyperglycemia, including ventricular hypertrophy and dysfunction associated with myocardial lipid accumulation, which is exacerbated by a high fat (HF) diet enriched in long-chain FFA (LCFA) [3], [6], [10]. Therefore, in this study, diabetic db/db mice and MHC-PPARα mice were characterized and administrated with or without APS, with C57 wide- type mice as normal control. To this end, we found that therapy with APS could well reverse the PPARα-mediated myocardial lipotoxicity in the pathogenesis of diabetic cardiomyopathy.

## Materials and Methods

### Animal Experiments

Male homozygous B6.Cg-m+/+ Leprdb/J (*db/db*) mice and male MHC-PPARα transgenic mice (404-3) in C57BL/6J background were obtained from the Jackson Laboratory (Bar Harbor, USA). All db/db mice were fed with standard chow, while all MHC-PPARα transgenic mice were fed with a HF diet composed of 43% of calories from fat containing triacylglyceride (TAG) composed of LCFA (16∶0 AND 18∶1) till the end of experiment. Five-week-old db/db mice and MHC-PPARα transgenic mice were both administrated with or without APS 2.0 g/kg/d until 20 weeks of age. Age-matched male C57BL/6J wild-type (Wt) mice (Laboratory Animal Unit of Fudan University School of Medicine) were fed with standard chow as the normal control. All mice were housed within Fudan University School of Medicine Animal Facility with free accesss to food and water. Mice were anesthetized with diethyl ether before sacrifice. Blood samples were obtained from the heart directly. The hearts were excised and ventricle tissues were frozen in liquid nitrogen. All animal experimental protocols were approved by the Animal Ethics Committee of Fudan University School of Medicine.

### Serum Chemistry

Serum levels of glucose, insulin, triacylglyceride (TAG) and free fatty acid (FFA) were determined using colorimetric assays by the Clinical Medical Research Unit Core Center at Huashan Hospital, Fudan University.

### Echocardiographic Studies

Before sacrifice, transthoracic M-mode and 2D echocardiographs were performed on conscious mice by using the Acuson Sequoia 256 Echocardiography System (Acuson, Mountain View, USA) with methods for measurements and chamber size using M-mode [16].

### Analyses of Myocardial TAG

After harvest, a midventricular slice of myocardium was snap-frozen in a cryomold containing OCT for sectioning. To detect neutral lipid, frozen sections were stained with oil red O and counterstained with hematoxylin. The quantitative analysis of myocardial TAG species by electrospary ionization mass spectrometry (ESI/MS) was performed by the Morphology Core of Fudan University School of Medicine [17].

### Cardiac Fuel Utilization Analyses

Basal rates of glucose and palmitate uptake in heart were assessed in awake mice by intravenously injecting 2-deoxy-D-[1-^14^C]glucose or [^14^C]palmitate (PerkinElmer Life and Analyticl Sciences, USA), heart and plasma samples were obtained accordingly for the measurement of 2-[^14^C] deoxyglucose-6-phosphate (DG-G-P) or [^14^C] incorporation into triglyceride [18].

Mouse working hearts were perfused with Krebs–Henseleit solution containing 5 mmol/L glucose, 10 µU/mL insulin, and 1.2 mmol/L palmitate. Myocardial FFA and glucose oxidation rates were determined by quantitative collection of ^3^H_2_O or^ 14^CO_2_ (PerkinElmer Life and Analyticl Sciences, USA) produced by the hearts perfused with buffer containing [9,10-^3^H]palmitate or [U-^14^C] glucose. [18].

### Quantitive Real-Time RT-PCR Analysis

Total RNA was extracted from cardiac tissues using TRIzol reagent (Invitrogen, Carlsbad, USA), and was subjected to reverse transcription using the Superscript first-strand cDNA synthesis system (Promega, USA) according to the manufacturer’s instruction (Table S1). The relative gene abundance was quantified by real-time PCR using GreenER™ qPCR Supermix (Invitrogen, Carlsbad, USA) as described previously [15]. The reactions were performed in an ABI 7000 sequence deletion system. The sequences of primers are listed as table S1. The relative gene expression was analyzed using the 2^(–ΔΔCt)^ method and normalized against 18S rRNA.

### Western Blot Analyses and Enzyme Activity Analyses

Western blot analyses were performed with cardiac whole-cell extracts as previously described [15] by using GAPDH as loading control (Sigma, St Louis, USA). All protein bands corresponding to targeted antibodies (Sigma, St Louis, USA) were scanned for quantification of protein expression levels. The densities of the bands in samples of the db/db mice with different treatment were normalized to the densities of the corresponding bands in the C57 wide-type samples. PPARα activities in myocardial tissue were also determined at the end of the experiment by RIA, and were measured using a modification of the procedure of Okunishi et al. (Peptide Institute Inc. USA).

### Statistical Analysis

All analyses were performed with the Statistical Package for Social Sciences version 14.0 (SPSS, Chicago.IL). Data are expressed as means ± S.E.M. Statistical significance between the two groups was determined by using the Student’s t-tests. In all statistical comparisons, a P value <0.05 was used to indicate a statistically significant difference.

## Results

### APS Rescued Cardiac Dysfunction in db/db Mice and MHC-PPARα Mice

**Figure 1:**
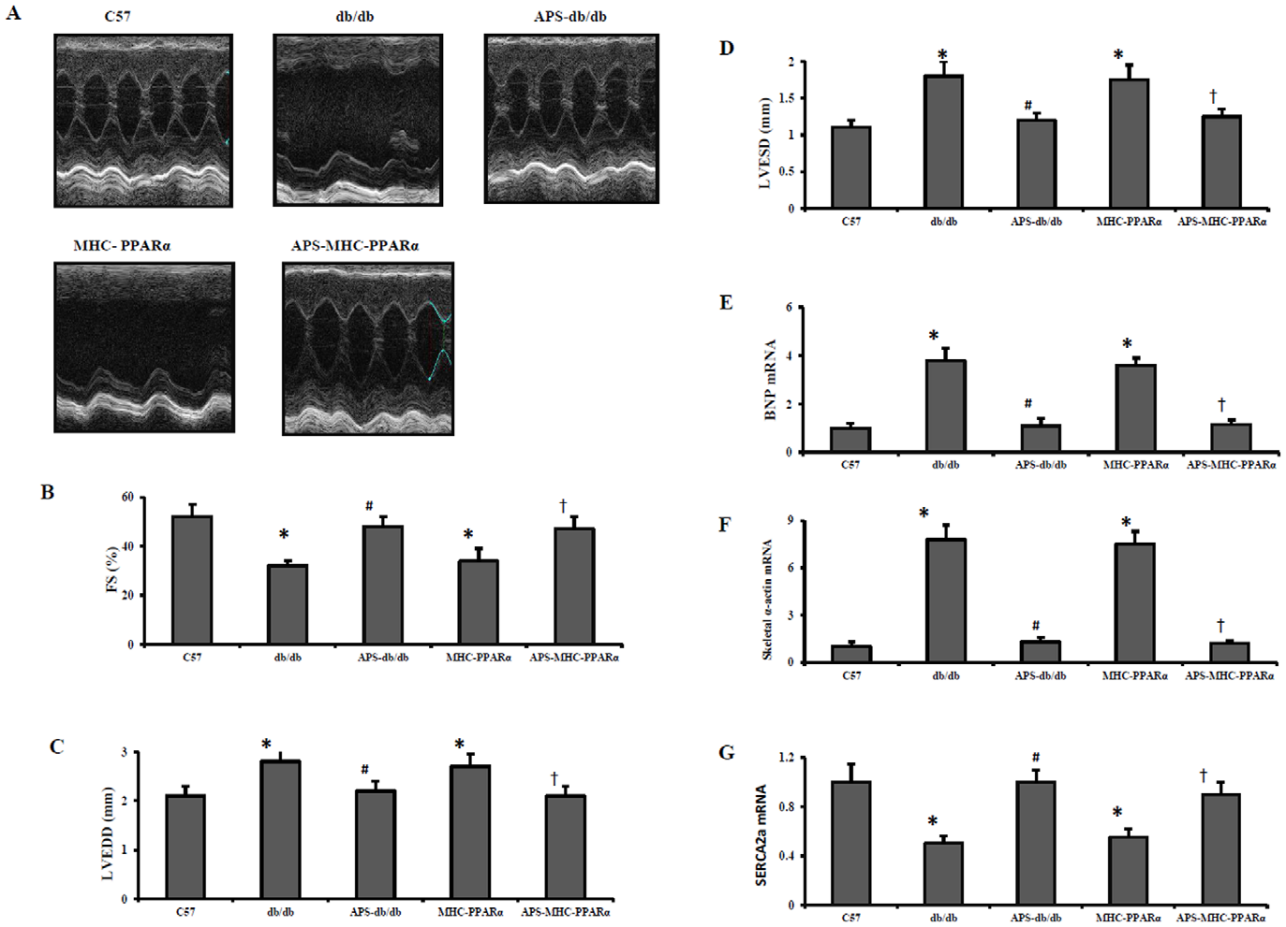
Echocardiographic properties and expression of cardiac dysfunction marker genes in hearts of mice. Echocardiographic studies were performed on db/db mice and MHC-PPARα mice with or without APS administration at the age of 20 weeks, with C57 wide-type mice as normal control. (A) Representative echocardiographic images; (B) fractional shortening (FS). Expression of cardiac dysfunction marker genes encoding BNP (C), Skeletal α-actin (D) and SERCA2a (E) was determined by real-time RT-PCR analysis. The relative gene expression of db/db mice and MHC-PPARα mice was normalized to the value of C57 wide-type mice, and expressed as fold change. All groups n = 8. Bars represent means±S.E.M. *P<0.05 vs. C57 mice, # *P*<0.05 vs. untreated db/db mice, † *P*<0.05 vs. untreated MHC-PPARα mice. BNP, brain-type natriuretic peptide; SERCA2a, sarcoplasmic/endoplasmic reticulum Ca^2+^ATPase 2a; MHC, myosin heavy chain; PPAR, peroxisome proliferator-activated receptor; APS, astragalus polysaccharides.

Diabetic cardiamyopathy is characterized by ventricular hypertrophy and diastolic dysfunction, finally leading to systolic ventricular dysfunction and overt congestive heart failure [19]. To determine the therapeutic effects of APS treatment on cardiac dysfunction in lipotoxic cardiomyopathy with or without diabetes, echocardiography was performed on both db/db mice and MHC-PPARα mice with or without APS treatment. As expected, both db/db mice and MHC-PPARα mice exhibited decreased reduced fractional shortening (FS) and the increased left ventricular (LV) chamber dilatation, as determined by measurement of systolic(LVISD) and diastolic (LVIDD) internal diameters, compare with C57 Wt controls (Figure 1). However, the reduction of FS in both db/db mice and MHC-PPARα mice were dramatically reversed by APS treatment, while the increase of LV chamber dilatation in db/db and MHC-PPARα mice was also inhibited after APS administration (Figure 1 and Table 1).

**Table 1:** Hemodynamics and cardiac function in mice.

	Wt	db/db	APS+db/db	MHC- PPARα	APS+MHC− PPARα
**HR (bpm)**	649±27	585±40	626±32	525±38	638±35
**LVPWd (mm)**	0.68±0.01	0.82±0.03*	0.72±0.03#	0.87±0.03*	0.73±0.02†
**IVSd (mm)**	0.70±0.01	0.84±0.04*	0.73±0.02#	0.85±0.04*	0.72±0.02†
**LVIDd (mm)**	3.2±0.02	3.6±0.04*	3.3±0.03#	3.7±0.04*	3.3±0.03†
**LVPWs (mm)**	1.10±0.06	1.42±0.02*	1.12±0.06#	1.45±0.03*	1.11±0.08†
**IVSs (mm)**	1.13±0.07	1.50±0.04*	1.16±0.03#	1.51±0.05*	1.14±0.04†
**LVIDs (mm)**	1.41±0.05	2.48±0.16*	1.64±0.10#	2.52±0.27*	1.63±0.09†

Values are mean±S.E.M. n = 8 each group.

* p<0.05 vs. wide type (Wt) mice;

# p<0.05 vs. untreated db/db mice;

† P<0.05 vs. untreated MHC-PPARα mice.

HR, heart rate; LVPWd/s, left ventricular posterior wall thickness at diastole or systole; IVSd/s, interventricular septal wall thickness at diastole or systole; LVIDd/s, left ventricular internal diameter at diastole or systole.

Moreover, the expression of hypertrophic growth marker genes encoding brain-type natriuretic peptide (BNP) and skeletal α-actin was induced in ventricle of both db/db mice and MHC-PPARα mice, while the expression of sarcoplasmic/endoplasmic reticulum Ca^2+^ATPase 2a (SERCA2a), acting as an endogenous regulator of pathological cardiac growth and decreasing in diabetic hearts, was reduced in db/db diabetic hearts and MHC-PPARα hearts, compared with those in C57 hearts (Figure 1). Meanwhile, the elevation of cardiac BNP and skeletal α-actin expression together with the down-regulation of SERCA2a in db/db diabetic hearts and MHC-PPARα hearts were all significantly normalized by APS treatment to the extent of C57 Wt controls (Figure 1).

**Figure 2:**
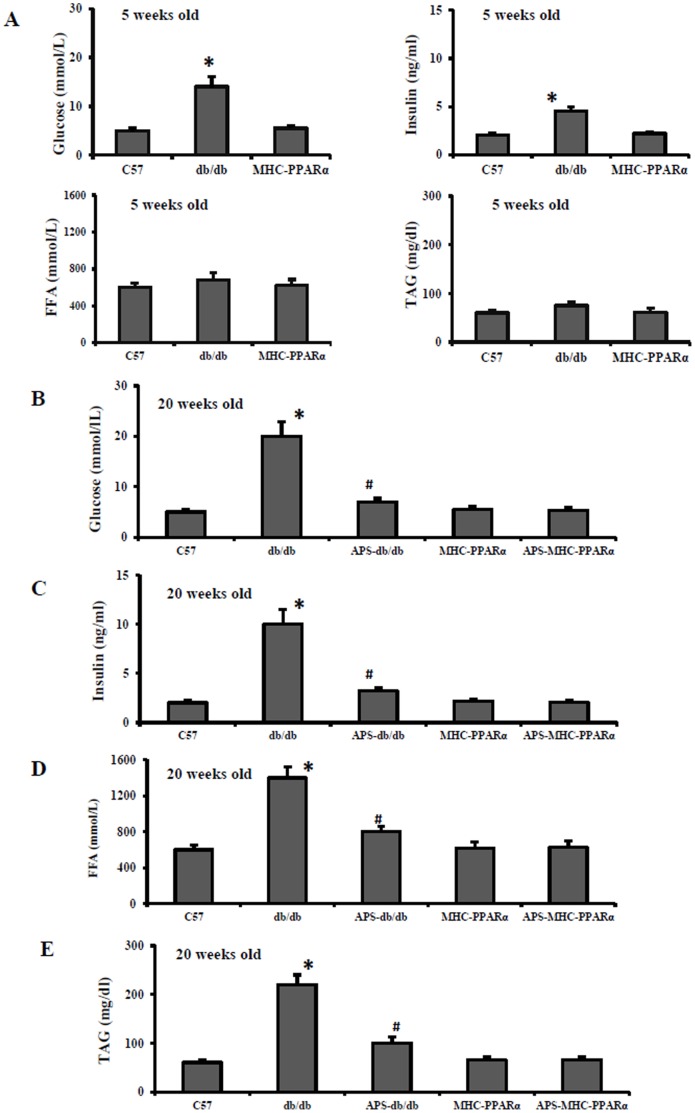
Serum biometric parameters of mice at different age. Serum glucose, insulin, nonesterified fatty acid (FFA) and triacylglyceride (TAG) were detected in db/db mice and MHC-PPARα mice with or without APS administration at the age of 5 weeks (A) and 20 weeks (B–E) respectively, with C57 wide-type mice as normal control. All groups n = 8. Bars represent means±S.E.M. *P<0.05 vs. C57 mice, # *P*<0.05 vs. untreated db/db mice, † *P*<0.05 vs. untreated MHC-PPARα mice.

On the other hand, although APS treatment had the significant systemic effect on the metabolic disorder in db/db mice, with decreases in the elevated levels of serum glucose, insulin, TAG and FFA, to the normal systemic metabolic state of MHC-PPARα mice (Figure 2), the rescue of cardiac dysfunction and abnormal expression of cardiac marker genes in APS-treated MHC-PPARα mice showed no difference from those in APS-treated db/db mice. Therefore, our finding demonstrated that APS treatment might rescue cardiac dysfunction in diabetes directly, independent of it effect on systemic metabolic disorder.

### APS Prevented Myocardial Lipid Accumulation in db/db Mice and MHC-PPARα Mice

**Figure 3:**
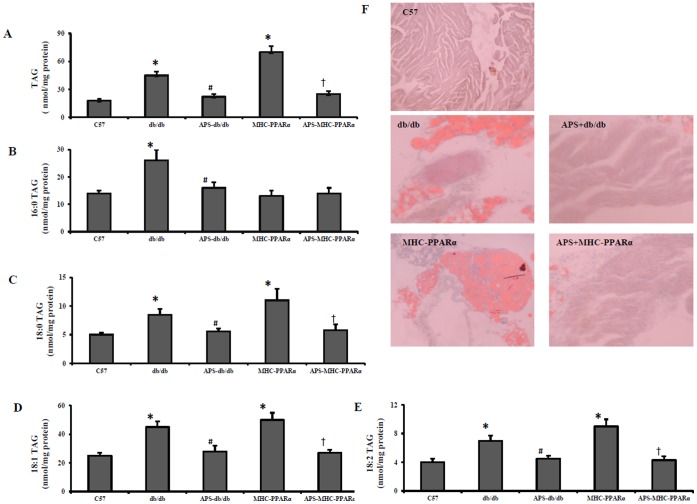
Intramyocardial lipid accumulation in mice. Myocardial triacylglyceride levels (A) in db/db mice and MHC-PPARα mice with or without APS administration at 20 week-old were determined by ESI/MS. Mean levels of TAG-associated fatty acid with chain length of 16∶0 (B), 18∶0 (C), 18∶1 (D), and 18∶2 (E) in the hearts of each group were shown respectively. (F) representative images of myocardium stained with oil red O. All groups n = 8. Bars represent means±S.E.M. *P<0.05 vs. C57 mice, # *P*<0.05 vs. untreated db/db mice, † *P*<0.05 vs. untreated MHC-PPARα mice.

Cardiomyocyte lipotoxicity is proved to be contribute to diabetic cardiomyopathy [20]. To determine the role of APS treatment in the myocardial lipid accumulation that occurs in diabetic cardiomyopathy, the TAG content in in db/db hearts and MHC-PPARα hearts was analyzed. As expected, measurement of cardiac TAG content exhibited a large accumulation of neutral lipid in both db/db hearts and MHC-PPARα hearts compare with those in Wt controls, while this lipid accumulation in db/db and MHC-PPARα hearts was markedly attenuated by APS treatment (Figure 3). Further ESI/MS profiling of myocardial TAG species revealed that APS treatment mainly reversed the elevation of TAG-associated fatty acid with chain length of 18∶0, 18∶1 and18∶2 in db/db hearts and MHC-PPARα hearts, compared with those in WT controls (Figure 3).

Furthermore, although APS treatment significantly reversed the serum hyperlipidemia in db/db mice to the normal serum lipid levels of MHC-PPARα mice, there was no difference in the evident TAG accumulation between APS-treated db/db hearts and APS-treated MHC-PPARα hearts (Figure 2 and 3). Collectively, these data showed that treatment with APS could prevent long chain fatty acid accumulation in diabetic hearts independent of it effect on systemic hyperlipidemia.

### APS Normalized Cardiac Glucose and FFA Metabolic Derangements in db/db Mice and MHC-PPARα Mice

**Figure 4:**
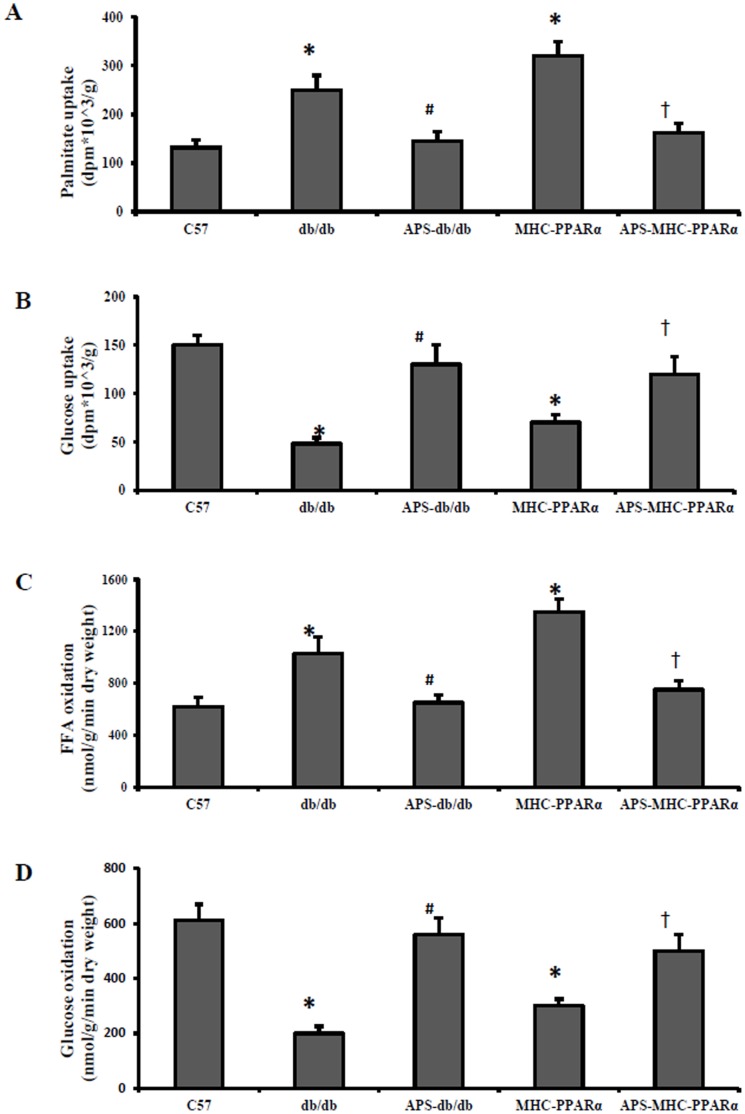
Myocardial fuel untilization in mice at the age of 20 weeks. Basal cardiac palmitate uptake (A) and glucose uptake (B) were measured in hearts from awake db/db mice and MHC-PPARα mice with/without APS at the age of 20 weeks, with C57 mice as normal control. Free fatty acid oxidation (C) and glucose oxidation (D) were assessed in isolated working hearts from db/db mice and MHC-PPARα mice with/without APS at the age of 20 weeks, with C57 mice as normal control. All groups n = 8. Bars represent means±S.E.M. *P<0.05 vs. C57 mice, # *P*<0.05 vs. untreated db/db mice, † *P*<0.05 vs. untreated MHC-PPARα mice.

Diabetic cardiomyopathy exhibits myocardial fuel utilization abnormalities with increased FFA import/oxidation and reduced glucosed utilization [21]. To assess the metabolic correlates of the functional rescue conferred by APS treatment in diabetes, myocardial substrate uptake and oxidation rates were determined in the isolated working hearts and awake mice from db/db mice and MHC-PPARα mice. As predicted, the palmitate uptake rates and FFA oxidation rates were increased in both db/db hearts and MHC-PPARα hearts, while the uptake and oxidation rates of glucose were decreased accordingly in db/db mice and MHC-PPARα mice, compared with those of Wt hearts (Figure 4). Notably, the elevation of FFA uptake and oxidation rates in both db/db hearts and MHC-PPARα hearts was substantially reduced by APS treatment, whereas the reduction of cardiac glucose utilization in db/db hearts and MHC-PPARα hearts was consistently normalized by APS treatment to the extent of Wt controls (Figure 4). Moreover, although therapy with APS had evident influence on the systematic hyperglycemia and hyperlipidemia in db/db mice, the beneficial effect of APS treatment on the normalization of cardiac glucose and FFA metabolic derangements were the same in db/db hearts and MHC-PPARα hearts, indicating that APS might benefit cardiac substrate metabolism directly in diabetic cardiomyopathy.

### APS Influenced the Myocardial PPARα Regulatory Pathway in db/db Mice and MHC-PPARα Mice

**Figure 5:**
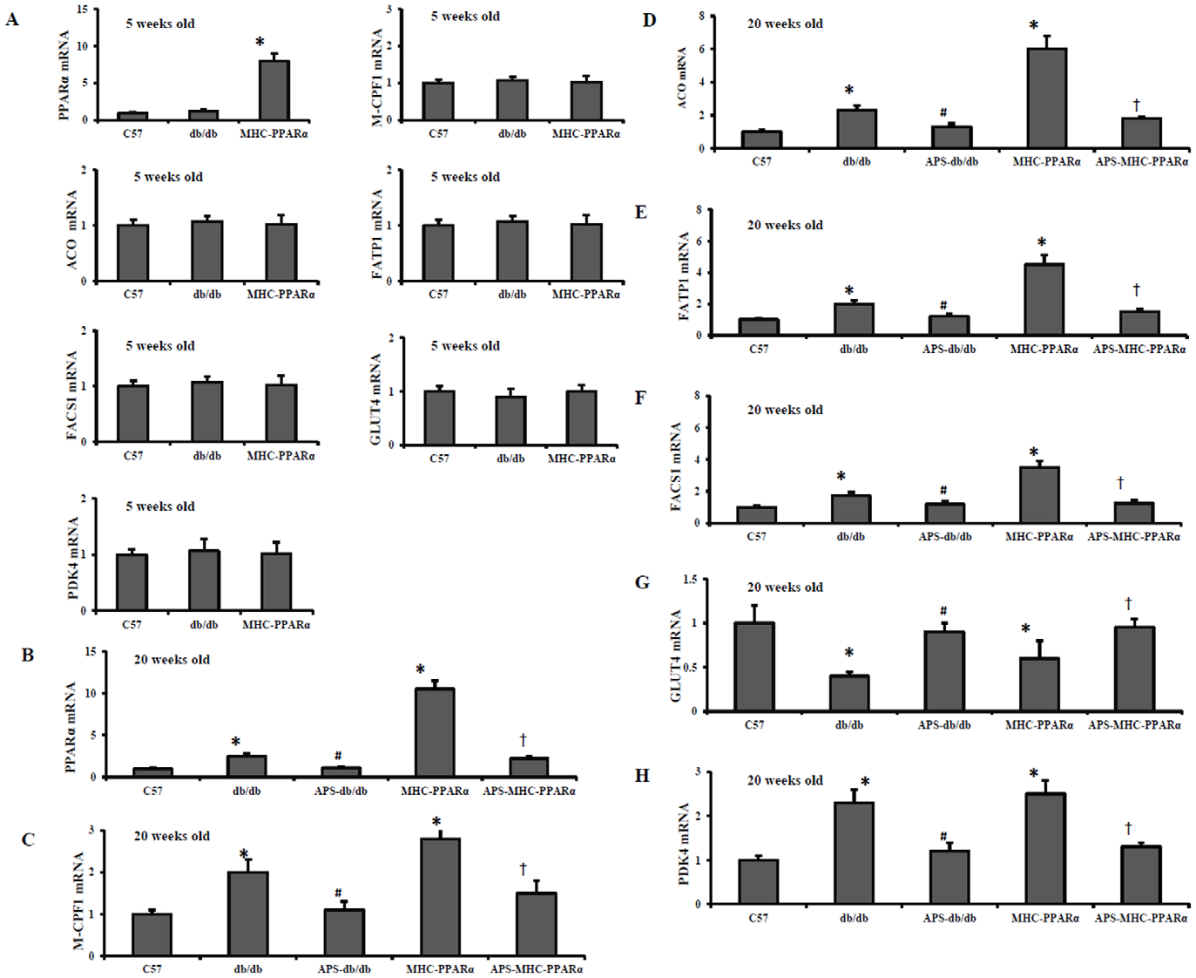
Gene expression of PPARα target genes in hearts of mice at different age. Cardiac tissues from db/db mice and MHC-PPARα mice with/without APS administration were detected for the gene expression at the age of 5 weeks (A) and 20 weeks (B-H) respectively, with C57 mice as normal control. Myocardial mRNA levels of PPARα target genes were determined by real-time RT-PCR analysis, encoding PPARα (B), M-CPF 1(C), ACO (D), FATP 1 (E), FACS 1(F), GLUT4 (G) and PDK4 (H). The relative gene expression of db/db mice and MHC-PPARα mice was normalized to the value of C57 wide-type mice, and expressed as fold change. All groups n = 6. Bars represent means±S.E.M. *P<0.05 vs. C57 mice, # *P*<0.05 vs. untreated db/db mice, † *P*<0.05 vs. untreated MHC-PPARα mice. M-CPF1, muscle-typecarnitine palmitoyltransferase 1; ACO, acyl-CoA oxidase; FATP 1, fatty acid trasport protein-1; FACS 1, fatty acyl-CoA synthetase 1; GLUT4, glucose transporter 4; PDK4, pyruvate dehydrogenase kinase 4.

**Figure 6:**
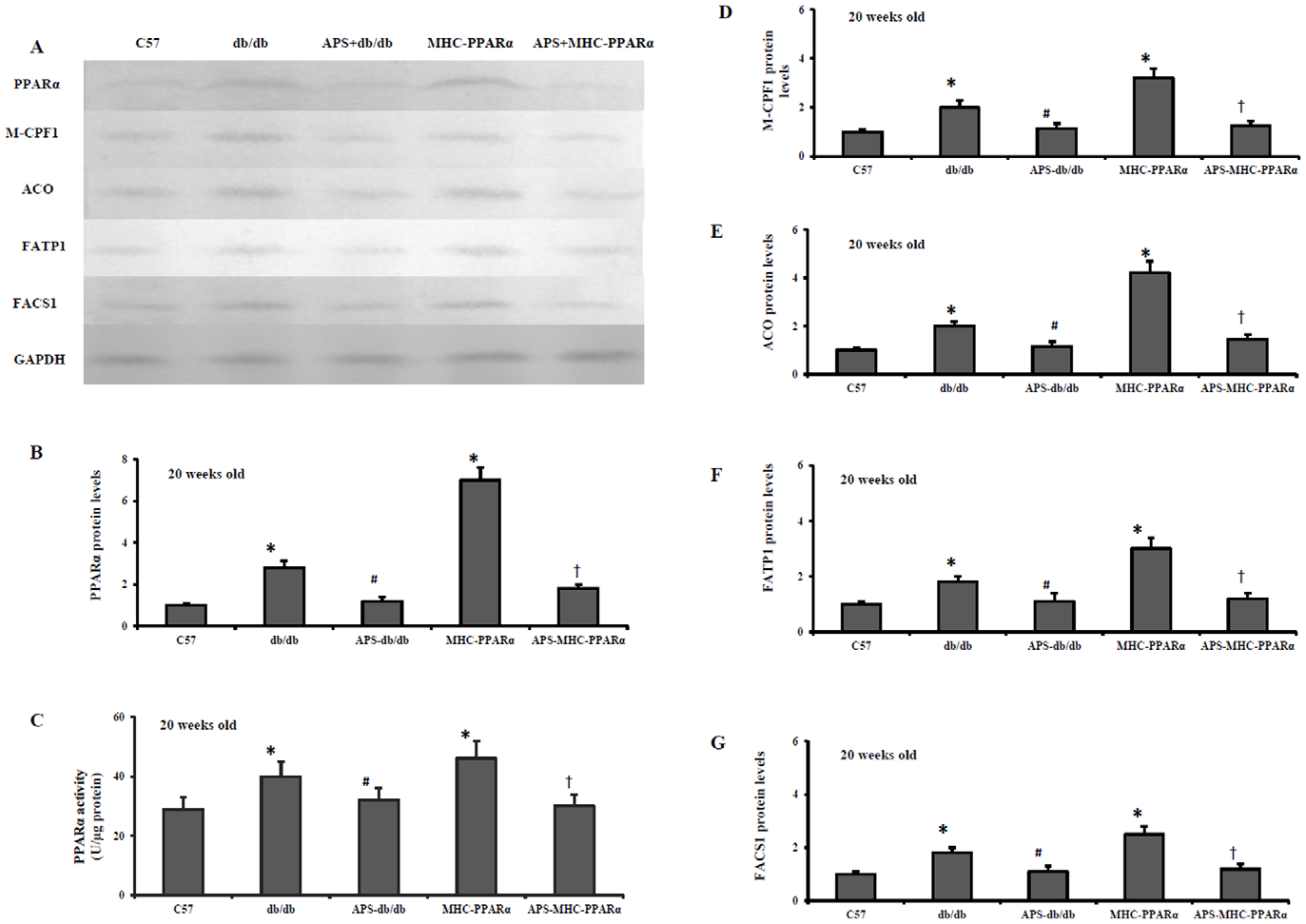
Protein levels of PPARα target genes in hearts of mice at the age of 20 weeks. Cardiac tissues from db/db mice and MHC-PPARα mice with/without APS administration were detected for the protein expression at the age of 20 weeks, with C57 mice as normal control. Myocardial protein levels of PPARα target genes were determined by Western Blot analysis. (A) Representative autoradiographs of PPARα target genes and GAPDH (loading control) using specific antibodies. Protein levels of PPARα target genes in myocardium, encoding PPARα (B), M-CPF 1 (D), ACO (E), FATP 1(F) and FACS 1(G). PPARα activities in myocardial tissue were also determined at the end of the experiment by RIA (C). All groups n = 8. Bars represented means±S.E.M., and were corrected for GAPDH signal intensity and normalized to the value of C57 wide-type mice. *P<0.05 vs. C57 mice, # *P*<0.05 vs. untreated db/db mice, † *P*<0.05 vs. untreated MHC-PPARα mice.

Activation of myocardial PPARα regulatory pathway mainly contribute to the pathogenesis of diabetic cardiomyopathy [19], [22]. The expression of genes involved in myocardial fuel utilization paralled the metabolic derangements in diabetic mice, with activated PPARα target genes involved in cardiac FFA oxidation, and downregulated myocyte glucose uptake and oxidation programs. As expected, the protein release of fatty acid transporter fatty acid trasport protein-1 (FATP 1) and fatty acyl-CoA synthetase 1 (FACS 1), served as LCFA transporters in hearts, was significantly elevated in db/db hearts and MHC-PPARα hearts compared with that of Wt controls, whereas the increase was reduced by APS treatment (Figure 5 and 6). In addition, the protein secretion of PPARα target genes involved in mitochondrial (muscle-type carnitine palmitoyl- transferase 1) and peroxisomal (acyl-CoA oxidase) fat oxidation pathways remained increased in db/db hearts and MHC-PPARα hearts compared with that of Wt controls, while the elevation was intensely reversed by APS administration (Figure 5 and 6). Consistently, the induction of gene expression of PPARα target genes involved in cardiac FFA import and oxidation in db/db hearts and MHC-PPARα hearts was substantially reversed by APS treatment at the same time, to the extent of mRNA expressions in Wt controls (Figure 5 and 6). With comparison, the gene expression of the glucose transporter GLUT 4 and the negative regulator of glucose oxidation PKD4 (pyruvate dehydrogenase kinase 4) was normalized in the db/db hearts and MHC-PPARα hearts by treatment with APS accordingly (Figure 5 and 6). To conclude, these finding suggested that APS treatment resulted in the normalization of myocardial PPARα regulatory pathway in both db/db mice and MHC-PPARα mice.

## Discussion

Growing evidence suggests that diabetic cardiomyopathy is common in diabetic individuals independent of hypertension and coronary artery disease [23]. The pathogenesis of diabetic cardiomyopathy is mainly associated with perturbations in cardiac energy metabolism. Fatty acids are the main source of energy production in normal hearts, whereas diabetes results in a marked reduction in cardiac glucose utilization and the heart relies almost exclusively on fatty acids (FFA) to generate energy [24]. Excess myocardial lipid accumulation due to an imbalance between fatty acid import and utilization, referred to as lipotoxicity, eventually promotes acquired forms of diabetic cardiomyopathies [25]. Multiple mechanisms contribute to the substrate switching that characterizes the diabetic heart. These include increased delivery of FFA, decreased insulin signaling, and activation of transcriptional pathways such as PPARα signaling network that regulates myocardial substrate use. The activation of PPARα gene regulatory pathway has been identified as a driver of the metabolic derangements contributing to lipotoxicity and cardiomyopathic remodeling in the diabetic hearts [19], [20].

PPARα, one of the PPAR isoforms, is recognized as a key regulator of myocardial fatty acid metabolism [19]. In the heart, PPARα activation induces expression of genes encoding nearly every step in the fatty acid utilization pathway including transport proteins to facilitate fatty acid uptake, acyl-coA synthetases for esterification of fatty acids to coenzyme A, fatty acid-binding proteins that facilitate delivery of fatty acids to different cellular compartments, mitochondrial carnitine system proteins that catalyze fatty acid transfer into the mitochondrion, every enzyme in the fatty acid β-oxidation pathway, and other accessory components of fatty acid metabolism such as uncoupling proteins [22], [26]. Transgenic mice with cardiac-restricted over-expression of PPARα (MHC- PPARα) exhibit the lipotoxic cardiomyopathic phenotype, including increased myocardial uptake and oxidation of FAs, myocyte TAG accumulation, reduced glucose utilization, characterized as metabolic and functional signatures of the diabetic heart. Mice with generalized deletion of PPARα (PPARα-null mice) demonstrate decreased fatty acid oxidation rates and concomitant increased glucose oxidation rates [22], [26]. In a murine model of pressure overload-induced cardiac hypertrophy, reactivation of PPARα with an agonist resulted in severe depression of cardiac power and efficiency [26], [27]. Studies in ob/ob and db/db mice also showed that [27], [28], while cardiac caloric excess became longstanding, activation of PPARα-mediated signaling increased the expression genes involved in FFA oxidation and FFA import such as CPT-1 and medium- and long- chain acyl CoA dehydrogenase, and FFA transporters such as FATP1, FACS1 and CD36, which contributed further to the metabolic changes in the heart; whereas the activation of PPARα increased the expression of PDK4, which further reduces glucose oxidation in myocardium. Therefore, the PPARα mediated lipotoxic cardiomyopathy is one of the important mechanisms in the pathogenesis of diabetic cardiomyopathy.

Astragalus (the root of Astragalus membranaceus, Huangqi), a traditional Chinese medicine, has been widely used in the clinical treatment of heart failure. Astragalus polysaccharides (APS) is a hydrosoluble component extracted from Astragalus and has been identified as its mainly bioactive ingredient [15]. Our previous study showed that treatment with APS rescued cardiac dysfunction and myocardial pathologic abnormality in diabetic animal models along with the improvement of the systemic derangement including hyperglycemia, hyperinsulinemia and hyperlipidemia [13]–[15]. However, it is still unclear whether APS could improve diabetic cardiomyopathy by preventing myocardial lipotoxicity, through a direct molecular mechanism independent of its effect on the systemic diabetic state. In an attempt to explore the underlying mechanism, diabetic db/db mice and MHC- PPARα mice were employed and treated with APS in our study. As speculated, the myocardial energy metabolic derangements leading to the deterioration of cardiac function in both db/db mice and MHC- PPARα mice were markedly inhibited by treatment with APS, with the reversal of developed LV chamber dilatation and systolic ventricular dysfunction, consistent with the reduction of activated gene expression of cardiac dysfunctional gene markers. In present study, the increased FFA utilization and decreased uptake and oxidation of glucose in diabetic hearts and MHC- PPARα hearts were also strikingly normalized by APS treatment with several lines of evidence to support. Firstly, APS threw a dramatic effect on the inhibition of enhanced TAG accumulation and the normalization of derangements of fatty acid composition in db/db diabetic hearts and MHC- PPARα hearts. Secondly, APS effectively reprogrammed the unbalance of substrate metabolism in db/db diabetic hearts and MHC- PPARα hearts with the decreased myocardial fatty acid utilization rates and the increased rates of glucose uptake and oxidation in myocardium. In addition, the increase of gene expressions and protein levels of PPARα target genes in db/db diabetic hearts and MHC- PPARα hearts, involved in fatty acid import and oxidation, was markedly reversed by APS administration. Finally, the abnormality of gene regulatory pathway referred to the derangements in glucose utilization observed in db/db diabetic hearts and and MHC- PPARα hearts was also normalized with APS treatment partly via PPARα. Notably, although the normal systemic metablic state in MHC- PPARα mice was not affected by treatment with APS, compared with the significant reversal of hyperglycemia and hyperlipidemia in db/db mice after therapy with APS, the prevention of cardiac dysfunction and myocardial lipotoxicity in the APS-treated MHC-PPARα mice is almost the same with that in the APS-treated db/db diabetic mice, which in turn further demonstrated that therapy with APS could rescue diabetic cardiomyopathy directly, independent of its influence on the systemic metabolic disorders. Summarily, our findings support the notion that treatment of APS has a beneficial effect on lipotoxic cardiomyopathy in diabetes through a mechanism mainly dependent on the modulation of PPARα regulatory pathway.

## Supporting Information

Table S1 **The sequences of primers used for real-time PCR analysis.**
(TIF)
