# Supplementary figures and images for: The Critical Role of Astragalus Polysaccharides for the Improvement of PPRAα-Mediated Lipotoxicity in Diabetic Cardiomyopathy
Source: PLoS One. 2012 Oct 1;7(10):e45541. doi: 10.1371/journal.pone.0045541 (PMC3462191; doi:10.1371/journal.pone.0045541)

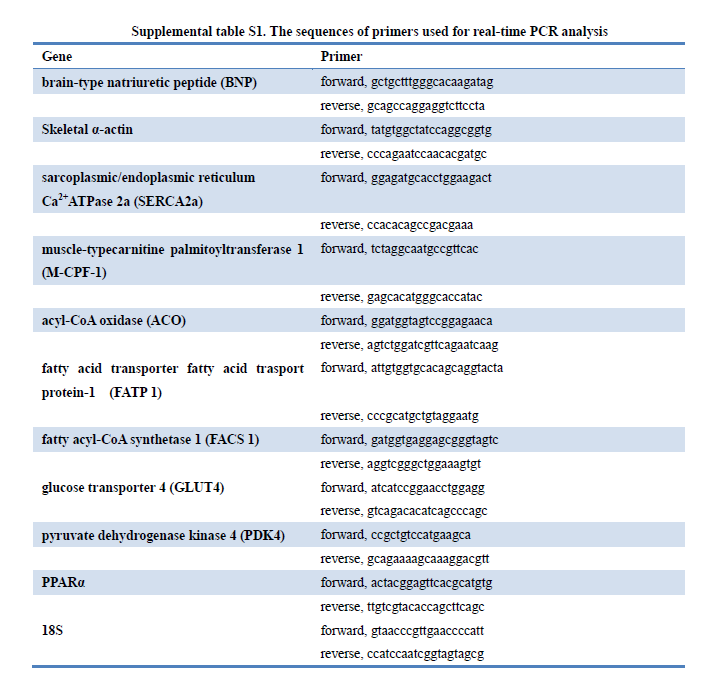

Supplement: Table S1 — The sequences of primers used for real-time PCR analysis. (TIF) [file pone.0045541.s001.tif]
